# In situ methane enrichment with vacuum application to produce biogas with higher methane content

**DOI:** 10.1007/s11356-024-33881-y

**Published:** 2024-06-07

**Authors:** Ozlem Sengur, Deniz Akgul, Baris Calli

**Affiliations:** Department of Environmental Engineering, Marmara University, Aydinevler, 34854 Istanbul, Türkiye

**Keywords:** Biogas upgrading, Carbon dioxide, Digestate, Biomethane, Waste activated sludge, Negative pressure

## Abstract

Sludge produced in sewage treatment plants is an important source of organic matter to be used in anaerobic digestion to produce energy-rich biogas. The biogas produced in anaerobic digesters has a critical impact on achieving carbon neutrality and improving energy self-sufficiency. After effective upgrading, biogas can be converted into biomethane with an increased CH_4_ content, resulting in a higher volumetric energy value. Upgrading biogas to biomethane thus not only improves its energy content but also broadens its potential uses. In this study, it was aimed at enrich CH_4_ by removing dissolved CO_2_ from the digestate using a vacuum, leveraging the solubility differences of gases in liquid. In this context, two digesters (R-T and R–C) were operated for 194 days, and the effect of vacuum on in-situ methane enrichment was investigated. The vacuum was only applied to the test reactor (R-T), and the CH_4_ percentage was increased from 63 to 87, 80, and 75% in the vacuum exposure time intervals of 30, 10, and 5 min, respectively. Extended durations were not tested, as the rate of enrichment decreased sharply after 30 min. The maximum energy requirement of a vacuum application was estimated at 0.124 kWh/m^3^ methane. Conversely, vacuum application did not cause any deterioration in biogas production, and the methane yields were similar in both reactors.

## Introduction

Anaerobic sludge digestion is a key process in sewage treatment plants that plays a significant role in achieving carbon neutrality and enhancing energy self-sufficiency. Anaerobic digestion (AD) of wastewater treatment sludge produces biogas as a renewable energy source. It is considered carbon–neutral because it captures and utilizes methane, which would otherwise be released into the atmosphere during the decomposition of organic matter. Methane is a potent greenhouse gas, and by capturing it, the treatment plant helps mitigate its impact on climate change. Because it typically contains 50–65% methane and 35–50% carbon dioxide, biogas has a relatively low volumetric heating value (21.5 MJ/Nm^3^) (Bansal et al. 2013). In recent years, incentives to upgrade biogas to a quality suitable for injection into the natural gas network and/or use as motor vehicle fuel have been increasing (Sarker et al. 2018).

Depending on the feedstock type and how the anaerobic digester is operated, the composition of biogas can vary. However, it is mainly composed of methane (CH_4_, 50–65%), carbon dioxide (CO_2_, 35–50%), and trace amounts of other gases such as water vapor, hydrogen sulfide, siloxanes, halogenated hydrocarbons, ammonia, oxygen, carbon monoxide, and nitrogen (Ryckebosch et al. 2011). The high CO_2_ content and other impurities in biogas are undesirable because they decrease the calorific value, and flame velocity (Budzianowski et al. 2017) and lead to a variety of operational issues such as deterioration in the pipeline and equipment. To eliminate them and increase the calorific value of the gas, biogas must be upgraded by removing CO_2_ and other impurities. The gas obtained in this way is called biomethane. Biomethane, with a methane content of 96–98%, can be used as vehicle fuel or injected into the natural gas grid and transmitted over long distances (Ryckebosch et al. 2011). Although there are different criteria in different countries for the biogas to be fed into the natural gas network, CH_4_ content is generally desired to be between 80 and 96% (Soreanu et al. 2011). Upgraded biogas, which has a higher CH_4_ content, emits less CO_2_ than that of fossil fuels (Papacz 2011).

The most commonly used methods for biogas upgrading (up to 96–98% CH_4_) are physical and chemical absorption, pressure and/or vacuum swing adsorption, membrane separation, cryogenic separation, and biological methane enrichment (Ryckebosch et al. 2011). The major obstacles associated with these technologies are substantial investment costs, methane loss during application, high chemical or water consumption, and operational difficulties in large-scale facilities (Fajrina et al. 2023). Recent studies focus on more cost-efficient biogas upgrading technologies, and hybrid technologies are recommended in which more than one process is used consecutively or together (Sahota et al. 2018).

Vacuum stripping is a separation process used in various industries to remove volatile components or contaminants from liquid streams. The method involves reducing the pressure in a vessel or column to create a vacuum that facilitates the evaporation and removal of volatile components from the liquid phase (Tao et al. 2024). In the field of environmental engineering, vacuum stripping is generally used for ammonia stripping (Zhang et al. 2017; Chen et al. 2021), sludge drying (Yan et al. 2009; Park et al. 2010; Avsar et al. 2021), or recovery of volatile fermentation products (Aydin et al. 2018; Okoye et al. 2022; Haroun et al. 2022). Nevertheless, there is no study in the literature that investigates the use of vacuum stripping for the removal of dissolved CO_2_ from digestate with the purpose of biogas upgrading. Vacuum stripping stands as a promising technology in biogas upgrading because it does not consume chemicals or water, does not require heat, and is characterized by its low energy requirement and compact structure. Therefore, most of the obstacles associated with conventional technologies may be eliminated by vacuum stripping.

In an anaerobic digester, the liquid (digestate) and gaseous (biogas) phases are in contact. Therefore, there is an equilibrium between the gases dissolved in the liquid and those in the gas phase. At 35 °C, CO_2_ is much more soluble in water (26.6 mmol/L) than CH_4_ (1.14 mmol/L) (Al Seadi et al. 2008). According to Henry’s Law, the solubility of gases decreases as temperature increases and pressure decreases. In addition, it is known that the volatilization rate of gases is directly related to their solubility in water (Tchobanoglous et al. 2014). Therefore, if the dissolved CO_2_ in the liquid phase is vacuum stripped, some more CO_2_ from the gas phase dissolves in the digestate.

In this study, it is hypothesized that applying a vacuum to the liquid phase (digestate) of an anaerobic digester will strip and exhaust dissolved CO_2_ from the reactor. The digestate, now depleted of dissolved CO_2_, is then expected to absorb additional CO_2_ generated during the non-vacuum period. Through periodic vacuum applications and dissolved CO_2_ removal, the methane content of biogas is expected to increase noticeably. This phenomenon is based on the fact that CO_2_ is much more soluble in digestate than CH_4_ (Bijos et al. 2022). To confirm this hypothesis, two lab-scale anaerobic digesters were set up. One digester was subjected to periodic vacuum applications to enrich methane in the biogas, leveraging the difference in solubilities of CO_2_ and CH_4_ in the digestate. To date, there are no studies in the literature on methane enrichment of biogas by periodic vacuum application during anaerobic sludge digestion, and this study is the first.

## Materials and methods

### Feed characteristics and operation of anaerobic digesters

**Fig. 1:**
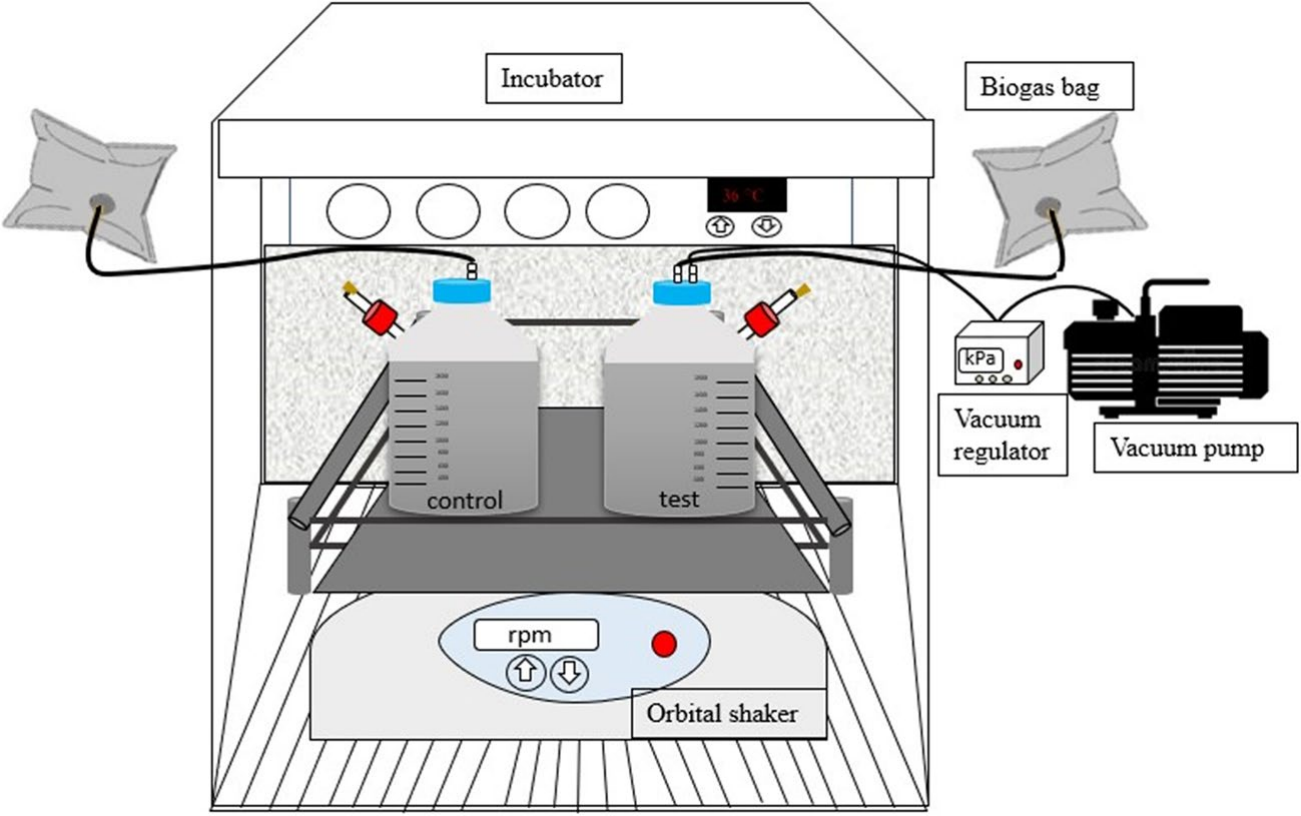
The set-up of anaerobic digesters

Two continuously stirred and daily fed lab-scale anaerobic digesters (control: R–C and test: R-T) with total and active volumes of 2 and 1.9 L, respectively, were operated for 194 days. Sludge retention time (SRT) and organic loading rate (OLR) were 20 days and 1.45 ± 0.29 g VS/L day, respectively. The reactors were continuously stirred on an orbital shaker (at 100 rpm) (PSU-20i Multi-functional Orbital Shaker) in an incubator at 36 ± 1 °C (WTW TS 606-G/2-i). The biogas produced was collected in 4-L aluminum foil gas bags. A schematic of the setup used is shown in Fig. 1.

The anaerobic digesters were fed with the dried waste-activated sludge (WAS) obtained from ISKI Pasakoy Domestic Wastewater Treatment Plant, Istanbul, Türkiye. The WWTP has a capacity of 200,000 m^3^/day and SRT of the plant varies between 10 and 15 days depending on the seasonal variations. WAS produced in Pasakoy WWTP is dried in a thermal sludge dryer.

The dried WAS was stored at room temperature in the laboratory, and large particles inside it were shredded before preparing the feed. 6.1 g of dried WAS was added to 90 g of water and mixed for 24 h in order to obtain a homogenous sludge feed. Withdrawing of effluents and feeding were accomplished using a syringe once a day. Characteristics of dried WAS and feed sludge can be found in Table 1. Inoculum was taken from an ongoing laboratory-scale mesophilic anaerobic digester, which had been fed with the same substrate (dried WAS) and OLR (1.5 g VS/L day) for 9 months (Sengur 2024). Following the start-up period, the test reactor was operated by applying vacuum for different periods of time.

**Table 1:** Characteristics of dried WAS, and feed sludge

	Dried WAS	Feed sludge
Total solid (TS; %)	95.7 ± 1%	6.1 ± 1%
Volatile solid (VS; %)	48.9 ± 1%	3.1 ± 1%
VS/TS	51.1%	51.1%
pH	–	7.3
Total COD (tCOD) (g O_2_/kg dry solid)	455.7 ± 10	
Total Kjeldahl nitrogen (TKN) (g N/kg dry solid)	52.3 ± 1.0	

Values are given as mean values ± standard deviation

### Vacuum application to test reactor

Initially, the test reactor (R-T) was operated by drawing digestate, applying vacuum to digestate in a separate vessel, and then feeding the vacuumed digestate back into the reactor. However, this operation was terminated due to air intrusion into R-T in each cycle. As a solution, we reduced the volume of headspace by increasing the active wet volume and started applying vacuum directly to the headspace of R-T once a day. After the start-up period (phase I, 40 days), R-T was subjected to vacuum (6 kPa) for 30 min every weekday in phase II. A double-stage oil-based vacuum pump with a capacity of 7.7 m^3^/h was used for this purpose. On day 67, the vacuum application was terminated for the next 35 days (phase III) to observe whether the reactor would return to initial steady-state conditions before the vacuum application. Next, the test reactor was subjected to vacuum once a day (weekdays) for 5 and 10 min in phases IV and V, respectively. Finally, the vacuum was terminated again until the end of the study (phase VI). The operating conditions of the reactors are given in Table 2.

**Table 2:** Operational control parameters in each phase

	Phase I		Phase II		Phase III		Phase IV		Phase V		Phase VI	
	Days 0–39		Days 40–66		Days 67–102		Days 103–142		Days 143–181		Days 182–194	
	R–C	R-T	R–C	R-T	R–C	R-T	R–C	R-T	R–C	R-T	R–C	R-T
EC (mS/cm)	9.28 ± 0.33	9.24 ± 0.35	9.37 ± 0.21	9.02 ± 0.18	9.36 ± 0.53	9.35 ± 0.48	8.66 ± 0.34	8.59 ± 0.20	8.99 ± 0.47	8.75 ± 0.30	10.04 ± 0.39	9.80 ± 0.29
CH_4_ (%)	62.7 ± 0.27	62.9 ± 0.26	63.1 ± 0.60	87.0 ± 2.22	63.4 ± 0.35	63.6 ± 0.51	63.4 ± 0.26	75.0 ± 1.47	63.4 ± 0.21	80.4 ± 1.34	63.2 ± 0.00	64.8 ± 0.82
CH_4_ (% increase in R-T over R–C)	–	–	–	37.6	–	–	–	18.3	–	26.8	–	–
CO_2_ (%)	37.3 ± 0.27	37.1 ± 0.26	36.9 ± 0.60	13.2 ± 2.22	36.6 ± 0.35	36.4 ± 0.51	36.6 ± 0.16	25 ± 1.47	36.6 ± 0.21	19.6 ± 1.34	36.8 ± 0.00	35.2 ± 0.82
Daily biogas production (mL/day)	498 ± 71	504 ± 69	1001 ± 113	793 ± 86	970 ± 142	928 ± 128	1053 ± 74	910 ± 57	1071 ± 72	801 ± 75	959 ± 229	932 ± 284
Daily CH_4_ production (mL/day)	313 ± 45	317 ± 43	602 ± 154	635 ± 166	614 ± 89	589 ± 80	663 ± 47	672 ± 43	680 ± 47	641 ± 60	606 ± 145	615 ± 180
CH_4_ yield (L CH_4_/g VS)	0.22 ± 0.03	0.22 ± 0.03	0.23 ± 0.02	0.24 ± 0.02	0.22 ± 0.02	0.21 ± 0.02	0.23 ± 0.01	0.23 ± 0.02	0.23 ± 0.02	0.22 ± 0.02	0.21 ± 0.03	0.20 ± 0.01
TAN (mg/L)	1234 ± 68	1246 ± 25	1238 ± 37	1243 ± 33	1330 ± 62	1321 ± 22	1284 ± 23	1284 ± 17	1283 ± 23	1300 ± 21	1342 ± 33	1381 ± 28
FAN (mg/L)	32 ± 2	31 ± 2	35 ± 3	145 ± 20	34 ± 2	37 ± 9	35 ± 2	68 ± 4	35 ± 1	100 ± 8	37 ± 2	39 ± 2
sCOD (mg/L)	1931 ± 120	1978 ± 106	2145 ± 160	2261 ± 163	2056 ± 133	2236 ± 95	2017 ± 88	2118 ± 89	1983 ± 60	2271 ± 62	2155	2402
VFA (acetic acid; mg COD/L)	143 ± 57	115 ± 60	180 ± 83	175 ± 79	166 ± 12	225 ± 29	150 ± 12	172 ± 16	111 ± 53	199 ± 123	172 ± 7	400 ± 36
TS (%)	4.19 ± 0.28	3.84 ± 0.27	4.17 ± 0.40	4.45 ± 0.28	4.05 ± 0.42	4.16 ± 0.34	4.77 ± 0.00	4.67 ± 0.00	4.36 ± 0.34	4.45 ± 0.01	3.94 ± 0.47	4.29 ± 0.24
VS (%)	1.99 ± 0.12	1.84 ± 0.11	1.93 ± 0.16	2.05 ± 0.12	2.00 ± 0.09	1.96 ± 0.15	2.22 ± 0.00	2.19 ± 0.00	2.04 ± 0.15	2.06 ± 0.00	1.87 ± 0.19	2.03 ± 0.07

Values are given as mean values ± standard deviation

*EC*, electrical conductivity; *CH*_*4*_, methane; *R-T*, test reactor; *R–C*, control reactor; *CO*_*2*_, carbon dioxide; *VS*, volatile solid; *TAN*, total ammonia nitrogen; *FAN*, free ammonia nitrogen; *sCOD*, soluble chemical oxygen demand; *VFA*, volatile fatty acids; *COD*, chemical oxygen demand; *TS*, total solid

In preliminary vacuum tests, it was determined that digestate boils below 5 kPa at 36 °C. To avoid water loss and consequent energy loss due to evaporation, the pressure in the headspace of the test reactor during vacuum applications was kept at about 6 kPa using a regulator. Following each vacuum application, the pressure in the headspace was equalized with the biogas stored in the gas bag, returning to atmospheric pressure. Therefore, the daily amount of biogas produced was determined by summing the volume utilized for pressure equalization in the headspace with the volume of gas collected in the gas bag over the previous 24 h.

### Analytical methods

The pH and electrical conductivity (EC) values were measured every day using a bench-top portable multimeter (WTW 3410 IDS). The soluble chemical oxygen demand (sCOD) was measured with the closed reflux colorimetric method (SM 5220-D) found in standard methods (APHA et al. 2023). For the sCOD analyses, the samples were filtered through a 0.45-μm syringe filter. Total ammonia nitrogen (TAN) concentrations were analyzed colorimetrically (SM 4500-NH3-A) (WTW 6100). The total solid (TS) (SM 2540-B) and volatile solid (VS)(SM 2540-E) concentrations were measured according to the standard method (APHA et al. 2023).

Volatile fatty acids (VFA) which are acetic acid, propionic acid, isobutyric acid, butyric acid, isovaleric acid, valeric acid, and caproic acid were determined by a gas chromatography (GC) instrument (Shimadzu, GC-2014) with a flame ionization detector (FID). In the VFA measurement method, helium (27.4 mL/min) was used as the carrier gas, and nitrogen as the makeup gas. In total, 1 μL filtered and diluted sample with 1% phosphoric acid solution was transferred to the capillary column (Stabilwax DA Fused Silica, 30 m × 0.25 × 0.5 µm) with the help of an autosampler. The injection port and detector temperatures were 250 and 260 °C, respectively. After the oven temperature was kept at 80 °C for 1 min, it was increased to 150 °C with an increase of 10 °C/min and kept constant at 150 °C for 3 min, and finally, it was increased to 200 °C with an increase of 10 °C/min and kept constant at 200 °C for 2 min. The analysis time was 18 min in total (Bayrakdar et al. 2017).

The volume of the biogas inside the biogas collection bags was measured daily using a gasometer based on the water displacement principle. The CO_2_ and CH_4_ content of the produced biogas was analyzed once a week in a gas chromatography device (Shimadzu, GC2014-NGA1) with a double thermal conductivity detector (TCD) and two different columns connected by a pneumatic valve system. The system kept O_2_, N_2_, and CH_4_ gases in the first column (Restek, Molesieve 13 × , 45/60, 9 ft × 1/8 in × 2 mm) and CO_2_ and H_2_S gases in the second column (Restek, HayeSep N, 60/80, 7 ft × 1/8 in × 2 mm). This allows the biogas composition to be analyzed with a single injection. Argon was used as the carrier gas, and dry air was used in the pneumatic valve system. The injection port and detector are both set to 150 °C. After the columns were kept at 40 °C for 4 min, the furnace temperature was increased to 65 °C with an increase of 5 °C/min. At this temperature, it was kept for 9 min and increased to 110 °C with an increase of 10 °C/min. The total duration of the analysis is 22.5 min (Molaey et al. 2018).

### Data analysis

The increase in CH_4_% and removal % of CO_2_ was found by Equations (Rasi et al. 2008) (1) and (2) for each phase, respectively.

1 $$\text{The increase in CH}_4 ({\%}) = \frac{{(\text{T}-\text{CH}}_{4})-({\text{C}-\text{CH}}_{4})}{{\text{C}-\text{CH}}_{4}}\times 100$$

2 $${\text{CO}}_{2}\text{ removal }\left({\%}\right)= \frac{{(\text{C}-\text{CO}}_{2})-{(\text{T}-\text{CO}}_{2})}{{\text{C}-\text{CO}}_{2}}\times 100$$

where T-CH_4_ and C-CH_4_ are the CH_4_ percent of R-T and R–C, respectively, and T-CO_2_ and C-CO_2_ are the CO_2_ percent of R-T and R–C, respectively.

The results were demonstrated as the mean values with a standard deviation using the Microsoft Excel program. For the statistical analyses, a paired *t* test was performed. A *p* value less than 0.05 was considered statistically significant.

## Results and discussion

### Effect of vacuum application on CH_4_ content of biogas

**Fig. 2:**
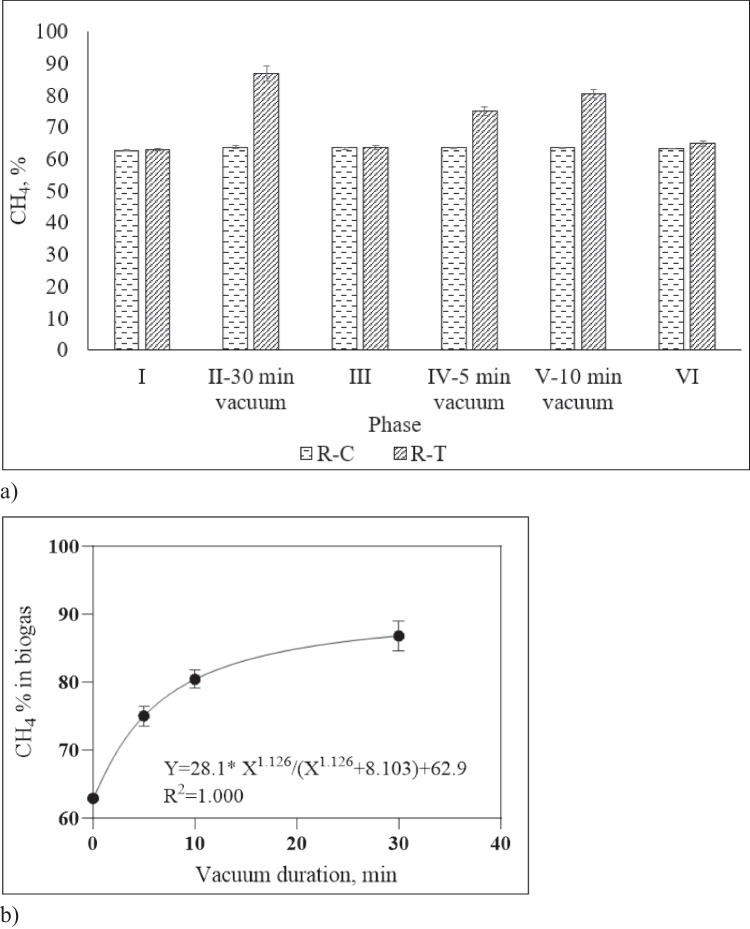
**a** Methane percentages of the anaerobic digesters at different phases; **b** CH_4_ percent of biogas measured in R-T at different vacuum durations (each data point represents average values, and error bars represent standard deviation of replicates obtained during each phase)

In the start-up period (Phase I), the control (R–C) and test (R-T) reactors were operated under the same conditions for 39 days until stable biogas production. A summary of the results in each phase can be seen in Table 2. In phase I, both reactors produced similar amounts of CH_4_ and the difference between average daily CH_4_ production was statistically insignificant (*t* test, *a* = 0.05, *p* = 0.75 > 0.05 for µ1 = µ2). In phase II, R-T was operated by applying a 30-min vacuum (6 ± 0.1 kPa) directly to the headspace of the reactor once a day. The highest methane enrichment was observed in this phase (phase II) and the CH_4_ content of biogas in R-T increased from 63.1 ± 0.60% to 87.0 ± 2.22%. In phase III, daily vacuum application was terminated to observe whether the CH_4_ content of biogas dropped to its previous level, and both reactors were operated under the same conditions for 35 days between days 67 and 102. In phases IV and V, R-T was subjected to 5 and 10 min of daily vacuum application, resulting in 75.0 ± 1.47 and 80.4 ± 1.34% CH_4_ in biogas, respectively (Fig. 2a).

The long-run results of vacuum-based continuous CH_4_ enrichment tests show that there was a non-linear correlation between the CH_4_ content of biogas (Y) and the duration of vacuum application (X) (Fig. 2b). This correlation was expressed by the following empirical formula (Eq. 3).

3 $$Y=\frac{28.2*{X}^{1.126}}{{X}^{1.126}+8.103}+62.9$$

The increase in the CH_4_ content of biogas produced in vacuum-applied R-T was attributed to the differences in the solubilities of CH_4_ and CO_2_ gases in the digestate (Bijos et al. 2022). At 35 °C and 1 atm, the solubilities of H_2_, CH_4_, CO_2_, H_2_S, and NH_3_, the gases present in biogas, in water are 0.749, 1.14, 26.6, 82.2, and 20,600 mmol/L, respectively (Al Seadi et al. 2008; Cao et al. 2021). Notably, CO_2_ is approximately 23 times more soluble than CH_4_ at pH 7 and 35 °C, resulting in solubilization of more CO_2_ in the digestate compared to CH_4_. Consequently, although they are produced in stoichiometrically similar amounts during AD, CH_4_ is always present at higher percentages in biogas compared to CO_2_ in a properly operated anaerobic digester (Bywater et al. 2022).

In this study, the vacuum was applied to R-T to strip CO_2_ dissolved in digestate, which was then exhausted from the reactor. Subsequently, the digestate, now depleted in dissolved CO_2_, reabsorbed some CO_2_ generated during non-vacuum applied periods. By repeating this vacuum application and CO_2_ removal process, the methane content in the biogas considerably increased in R-T. In summary, this study demonstrated that the in-situ methane enrichment through vacuum stripping, as developed and tested at a laboratory scale, can serve as an alternative to or integrated with conventional biogas upgrading technologies. It has been proven that this approach can successfully increase the CH_4_ content in biogas to over 87%.

During vacuum application, it is likely that some CH_4_ was also stripped from R-T along with CO_2_. However, considering that CH_4_ has approximately 23 times lower solubility than CO_2_, the CH_4_ lost during vacuum application was expected to be negligible. Additionally, although we have not used, biogas stripped from the liquid phase and then drawn from the headspace of the reactor during daily vacuum application of a maximum of 30 min can be enriched by passing it through a CH_4_ selective membrane or an alkaline scrubber. The resulting CH_4_-enriched biogas can be mixed with the main biogas stream ensuring no CH_4_ loss.

Only a very small part of the biogas produced in this system can be withdrawn during the vacuum period however, it can be returned to the biogas collection bag following a simple scrubbing applied to remove CO_2._ Separation of CO_2_ from biogas is a topic that has been studied many times in the literature, so it was not included in the scope of this article.

**Fig. 3:**
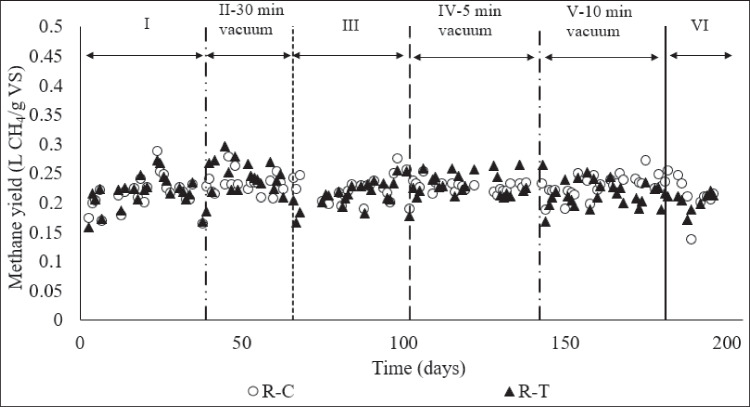
Methane yields of anaerobic digesters

In addition, contrary to some studies in the literature, our experimental results showed that applying a vacuum did not have a detrimental impact on the biological activity in R-T. In a similar study investigating the effect of vacuum on biogas production, it was reported that pure culture methanogens were not adversely affected for 10 days under 40 kPa vacuum at 35 °C, but experienced serious stress at 5 kPa (Kral et al. 2011). In our study, it was determined that short-term (max 30 min) daily vacuum application (6 kPa) did not adversely affect methane production, and the methane yields (CH_4_ produced per kg of VS) of vacuum-applied R-T and non-vacuumed R–C were quite similar (*t* test, *α* = 0.05, *p* = 0.00 > 0.05 for µ1 = µ2) (Fig. 3). The microbial populations in mixed anaerobic cultures as in this study, demonstrate a synergistic relationship, exhibiting greater robustness and resilience to environmental changes compared to pure cultures (Redl et al. 2017). Therefore, even though similar pressure values were tested in our study and in the studies using pure culture, contrary to the literature, no deterioration in the activity of methanogens was observed. This synergistic relationship explains why short-term cyclic vacuum application is not detrimental to methanogens and methane production.

### Effect of vacuum application on carbonate equilibrium and pH of the reactor

**Fig. 4:**
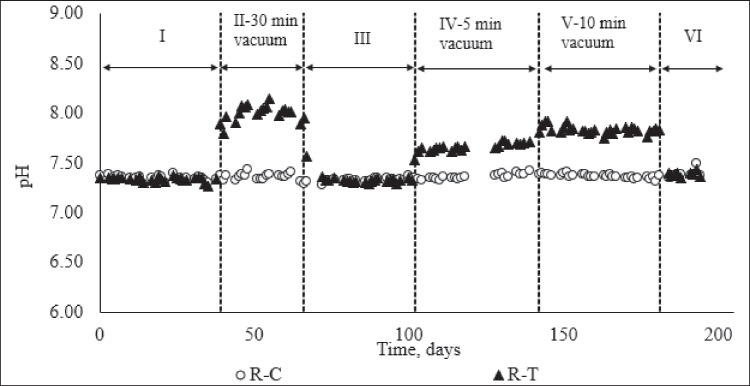
pH values of anaerobic digesters

The removal of CO_2_ by vacuum stripping also affected the pH values in R-T. The average pH value was 7.33 ± 0.02 at phase I in R-T, and it increased to 8.14 ± 0.16 after starting the vacuum application in Phase II (Fig. 4). The optimal pH range for AD is between 6.8 and 7.5 (Gonde et al. 2023). However, pH values higher than 7.5 in R-T did not adversely affect the performance of the reactor. Following the termination of the vacuum application, the pH of R-T decreased to the initial (pre-vacuum) value and equalized with the pH value of R–C at 7.3. The pH value differences between R–C and R-T in phases II, IV, and V were statistically significant (*t* test, *α* = 0.05, *p* = 0.00 < 0.05 for µ1 = µ2). There is an equilibrium between CO_2_ in the gas and liquid phases of an anaerobic digester, and this equilibrium significantly affects the pH of the digestate. When CO_2_ dissolves in digestate, it forms carbonic acid, influencing the acidity and, consequently, the pH in the digester. So, there is an inverse correlation between the CO_2_ content in biogas and the pH of the digestate: lower CO_2_ percentages in biogas correspond to higher pH in the digestate (Boyd 2000).

Electrical conductivity (EC) is a parameter showing the ionic strength of solutions. The dissolution of CO_2_ in the liquid phase of the anaerobic digester results in the formation of mainly bicarbonate and hydrogen ions, which increases the EC of the digestate (Aceves-Lara et al. 2012). EC was monitored in both reactors (R-T and R–C) to estimate how the removal of CO_2_ affects the carbonate equilibrium. It can be seen in Table 2 that during vacuum application, the EC values in R-T decreased slightly depending on the amount of CO_2_ removed, according to the following equilibrium (Eq. 4).

4 $${\text{CO}}_{2}\left(\text{aq}\right)+{\text{H}}_{2}\text{O}\rightleftharpoons {\text{H}}_{2}{\text{CO}}_{3}(\text{aq})$$

### Effect of vacuum application on ammonia concentration in the reactor

Ammonia, which is the unionized form of ammonium, is a potential inhibitor for AD. TAN levels of approximately 1700–1800 mg/L were entirely inhibitory in non-acclimatized inoculum. However, with acclimation, the inhibitory TAN levels could rise to as high as 5000 mg/L (Yenigün and Demirel 2013). However, in sewage sludge digesters, TAN concentrations are usually below inhibitory levels and thus did not pose any inhibition risk (Capson-Tojo et al. 2020). Likewise, the average TAN concentration was 1290 ± 33 mg/L in this study. Although many studies in the literature focus on vacuum-based ammonia stripping from the digestate, the conditions appropriate for ammonia stripping (at 6 kPa, temperature > 42 °C and pH > 9) were not tested in this study (Sengur et al. 2023). On the other hand, free ammonia nitrogen (FAN) values in R-T increased from 31 ± 2 mg/L to 145 ± 20 mg/L due to rising pH values as a result of vacuum application (Table 2). Although these FAN values were close to the 150 mg/L inhibition threshold reported in some literature, the CH_4_ yield of R-T was not negatively affected (Yenigün and Demirel 2013).

### Effect of vacuum application on sCOD and VFA concentrations in the reactor

sCOD and VFA are critical monitoring parameters used to evaluate the performance of anaerobic digesters operated under different conditions. VFA consisted almost exclusively of acetic acid and never exceeded 432 mg/L throughout the 194 days of operation. Because the anaerobic conditions could not be adequately maintained when feeding R-T for several consecutive days, the highest acetic acid and sCOD concentrations were observed in phase VI, the last period with vacuum application (Table 2).

When organic materials degrade through anaerobic digestion, VFAs are produced as intermediates during the acidogenesis step. Because acetogens and methanogens have a critical role in the consumption of VFAs, unfavorable conditions such as oxygen intrusion into an anaerobic digester and sudden changes in pH and temperature result in the accumulation of VFAs in AD (Wainaina et al. 2019; Yin et al. 2021).

The fact that VFA concentrations in R-T are relatively low and very similar to those in R–C is important evidence that the conversion of VFA to CH_4_ occurred smoothly in all phases and that vacuum application did not deteriorate the methanogenic activity in R-T. On the other hand, throughout the study, sCOD concentration was usually slightly higher in R-T than in R–C. The slight difference in COD was not reflected in biogas production, and similar CH_4_ yields were obtained in both reactors. A more detailed study is needed to evaluate the disintegration effect of vacuum application on digestate.

### Energy requirement

In this study, an oil-based, energy-inefficient benchtop vacuum pump with a capacity greater than that required for vacuum stripping in a 2-L anaerobic reactor was used. Therefore, the power consumption value of a full-scale vacuum-assisted evaporator with energy recuperation was used to more realistically estimate the energy requirement of the developed process. It is known that using mechanical vapor recompression (MVR) technology significantly reduces the energy requirement of evaporation. MVR systems offer high energy efficiency as the energy contained in the vapor is recycled for use in the main heat exchanger, significantly reducing energy consumption. It is reported that a typical MVR evaporator uses 15–45 kWh to evaporate 1 m^3^ of water (condensate) (ENVIDEST MVR FF 2024). Therefore, the power requirement of vacuum-based in situ methane enrichment was assumed to be 45 kWh/m^3^ of water evaporated. Because only a negligible amount of water (max 1 mL/L of digestate) evaporated under the conditions tested for CO_2_ stripping (6 kPa and 35 °C), the energy consumption was calculated as 3.63 kWh/m^3^-digester for 30 min/day of vacuum application. This value corresponds to 1.24% of the energy to be obtained from daily methane production. The results of the energy requirement analysis conducted for vacuum-based in situ CH_4_ enrichment are presented in Table 3.

**Table 3:** Energy requirement of in situ methane enrichment through vacuum application

	5 min/day	10 min/day	30 min/day
Water evaporated (mL/L of reactor)	> 1.0		
Energy consumption^a^ (Wh/L of water evaporated)	45		
Energy consumption(Wh/L of reactor)	0.04		
Daily biogas production (mL/L of reactor)	479	422	417
Methane percent in biogas (%)	75.0	80.4	87.0
Daily methane production (mL/L of reactor)	359	339	363
Daily energy generation^b^ (Wh/L of reactor)	3.59	3.39	3.63
Energy consumed for vacuum application (% of energy generated)	1.25%	1.33%	1.24%
Energy consumption of vacuum application per CH_4_ production (kWh/m^3^ CH_4_)	0.125	0.133	0.124

^a^Energy consumption value of mechanical vapor recompression (MVR) vacuum evaporators (45 kWh/m^3^ of water evaporated) (ENVIDEST MVR FF 2024)

^b^Energy value of methane is assumed to be 10 Wh/L

With rising energy demands worldwide, biomethane production by methane upgrading technologies has gained importance. The main operational conditions and energy demands of several upgrading methods are summarized in Table 4. Most of the conventional methods are based on scrubbing technologies. These technologies can increase methane by up to 99%. However, the use of chemicals and other consumables significantly impacts operational costs and environmental sustainability. Another critical aspect of these upgrading technologies is their heating requirements. Chemical (amine) scrubbing and organic solvent scrubbing are applied at temperatures of about 100–180 and 55–80 °C, respectively, which increases the operational costs and environmental impacts. Another promising technology, membrane separation, requires several steps to achieve higher CH_4_ percentages, with CH_4_ losses of up to 10% during these steps (Baena-Moreno et al. 2019).

**Table 4:** Comparison of different methane upgrading methods with the in situ methane enrichment process

	Vacuum application (this study)	Water scrubbing	Chemical (amine) scrubbing	Organic solvent scrubbing	Pressure sawing adsorption	Membrane separation	Reference
Water demand	No	Yes	Yes	No	No	No	Baena-Moreno et al. (2019)
Consumables requirement	–	Antifouling agent, drying agent	Amine solution (hazardous, corrosive)	Organic solvent (non-hazardous)	Activated carbon (non-hazardous)	–	TUV (2012)
Pressure (abs; kPa)	6	500–1100	100	500–900	500–1100	700–900	Thrän (2012)
Methane content (%)	87	95–99	> 99	95–99	83–99	82–99	TUV (2012), Bekkering et al. (2010), and Allegue et al. (2012)
Energy demand (kWh/m^3^ biomethane)	0.13	0.46	0.27	0.49–0.67	0.23–0.46	0.14–0.43	TUV (2012), Bekkering et al. (2010), and Allegue et al. (2012)
Application temperature (°C)	36	–	100–180	55–80	–	–	TUV (2012)

By partially separating the CO_2_ from the methane gas stream due to the solubility difference, the energy content of the resultant gas is increased (Hayes et al. 1990). At 20 °C and 1 atm, the calorific value of biogas with a CH_4_% of 63 is 5390 kcal/m^3^ (22,566 kJ/m^3^) whereas the calorific value of biogas with a CH_4_% of 80 is 6376 kcal/m^3^ (26,695 kJ/m^3^) (Singh et al. 2017).

In a study exploring membrane separation for CH_4_ enrichment, an 82% CH_4_ concentration was attained with an energy demand of 0.14 kWh/m^3^ (Bekkering et al. 2010). In another study that tested pressure swing adsorption for CH_4_ enrichment, an 83% CH_4_ content was achieved with an energy demand of 0.23 kWh/m^3^ (Allegue et al. 2012). The in itu methane enrichment process developed and tested in this study increased the amount of CH_4_ in biogas up to 87.0% with only 30 min of daily vacuum application and a relatively lower energy requirement (0.124 kWh/m^3^ CH_4_) compared to conventional biogas upgrading processes having similar CH_4_ percentages. Our process is also compact and simple. The digestate itself was used as a scrubbing agent (absorber) without using additional chemicals, eliminating the need for extra scrubbing and regeneration units.

### Implications and limitations

Although the methane purification efficiency of vacuum stripping is insufficient for high-quality biomethane production, it can be used prior to upgrading technologies to reduce overall costs. The size and cost of upgrading systems are directly proportional to the amount of impurity to be removed, so costs increase with higher CO_2_ content in the biogas (Olugasa and Oyesile 2015). In addition, in situ vacuum application allows obtaining a CO_2_-rich gas, which can be used in various applications (Awe et al. 2017).

The efficiencies of conventional biogas upgrading technologies in the literature vary between 82 and 99% (Bekkering et al. 2010; TUV 2012). The demonstrated increase in methane content to over 87% through in situ methane enrichment via vacuum stripping suggests a promising alternative or complement to conventional biogas upgrading technologies. This approach not only improves the quality of biogas but also does so with relatively lower energy requirements through a simplified process. The ability to use the digestate itself as a scrubbing agent highlights the potential for economic and environmental benefits. However, the study acknowledges that this method may not be as efficient as conventional biogas upgrading methods, and further research is needed to optimize its effectiveness. Nonetheless, the ability to reduce overall costs by using in situ methane enrichment prior to biogas upgrading makes this approach a promising pathway for future biomethane production.

## Conclusion

In this study, the vacuum was applied to the headspace of an anaerobic sludge digester for varying durations, resulting in significant increases in the CH_4_ content of the biogas in all trials. Specifically, the CH_4_ percentage increased to 87.0 ± 2.2% with 30 min of vacuum application. This in situ methane enrichment process, developed and tested for the first time in this study, exhibits a low energy requirement compared to conventional biogas upgrading technologies based on membrane and adsorption. Unlike its counterparts, the process does not necessitate the use of chemicals or high temperatures and pressures. Our results demonstrate that the vacuum-based in situ methane enrichment system can serve as a cost-effective pre-treatment unit before employing other upgrading technologies, thereby significantly reducing operating costs. With optimized operating conditions, it may be feasible to produce biomethane with a purity comparable to that achieved with conventional technologies. In conclusion, the vacuum-based in situ methane enrichment system is poised to play an important role in achieving carbon neutrality and enhancing energy self-sufficiency in wastewater treatment plants in the future.

## Data Availability

The datasets used and/or analyzed during the current study are available from the corresponding author upon reasonable request.
